# Nonconsensual Dissemination of Sexual Images Among Adolescents: Associations With Depression and Self-Esteem

**DOI:** 10.1177/08862605231165777

**Published:** 2023-04-19

**Authors:** Beatrice Sciacca, Angela Mazzone, Magnus Loftsson, James O’Higgins Norman, Mairéad Foody

**Affiliations:** 1 DCU Anti-Bullying Centre, Dublin City University, Dublin, Ireland; 2 FRIENDS, Research and Development, Stockholm, Sweden; 3 School of Psychology, National University of Ireland, Galway, Ireland

**Keywords:** adolescents, sexting, nonconsensual dissemination, technology-facilitated sexual violence, depression, self-esteem

## Abstract

The nonconsensual dissemination of sexual images is a form of image-based sexual abuse that is relatively common among adolescents. However, literature on this issue with adolescent samples is relatively scarce. Therefore, this study is aimed at investigating how this phenomenon varies based on gender and sexual orientation, along with its association with depression and self-esteem. Participants were 728 secondary school students in Sweden (50.4% girls and 46.4% boys; 14.4% lesbian, gay, bisexual [LGB+]) aged from 12 to 19 years (*M* = 14.35, *SD* = 1.29). A survey was administered during school hours, including a measure assessing the nonconsensual dissemination of sexual images, the short version of the Moods and Feelings Questionnaire, and the Rosenberg’s Self-Esteem Scale. Results showed that LGB+ participants were more likely to indicate that they were victimized compared to their heterosexual peers, while no differences were observed for gender. Being the target of nonconsensual dissemination of sexual images was positively associated with depression, whereas no significant associations were yielded for self-esteem. Based on the findings from this study, we suggest raising adolescent awareness in relation to the nonconsensual dissemination of sexual images as a form of sexual abuse that can have detrimental effects on those who are targeted. Such educational programs should be inclusive of sexual minority adolescents, as they are at a particular risk of being the target of nonconsensual dissemination of sexual images. Psychological support should be provided to the targets of this form of abuse, through both school and online counseling. Future research should adopt longitudinal designs while recruiting diverse samples.

## Introduction

Adolescents’ communications and interactions often take place online, including when it comes to sexuality. The act of sending, receiving, and forwarding of sexual texts and pictures via technology is a phenomenon known as sexting (Klettke et al., 2014). Such behavior is increasingly recognized by scholars as a normal part of adolescents’ sexual development (Symons et al., 2018). As such, sexting might allow inexperienced adolescents to experiment with sexuality in a way that they feel comfortable with (Van Ouytsel et al., 2017). It could also be a way to flirt with a love interest or to maintain a romantic relationship (Dodaj et al., 2022; Van Ouytsel et al., 2017). Furthermore, adolescents might produce sexual pictures to obtain peers’ validation about their body and thus increase their self-confidence (Dodaj et al., 2022). Hence, sexting could be a way for adolescents to build intimacy and explore their sexuality (Hasinoff, 2015). Such positive outcomes are related to consensual sexting, whereby all the parties involved agree to the exchange of sexual pictures. However, sexting may also occur without the consent of one of the parties involved. Nonconsensual sexting, also referred to as “aggravated,” is a form of sexting that involves criminal or abusive elements, such as pressuring someone to sext, threatening someone with negative consequences if they do not sext, sending sexual pictures to nonconsenting recipients, or sharing someone’s sexts without their knowledge and permission (Harper et al., 2021; Wolak & Finkelhor, 2011). This work will focus on the latter, that is the nonconsensual dissemination of sexual images, defined as the sharing of a sexual picture or video without the consent of the person depicted (Walker & Sleath, 2017).

### Terminological Issues

We chose to use the terms “nonconsensual dissemination of sexual images” instead of the commonly used term “revenge porn,” as the use of the latter has been suggested to be problematic (DiTullio & Sullivan, 2019; Gassó et al., 2021; Henry et al., 2019; Maddocks, 2018; Walker & Sleath, 2017). “Revenge porn” refers to an instance whereby an individual publicly shares the private sexual pictures of an ex-partner without their consent, as a form of revenge (Gassó et al., 2021; Henry et al., 2019; Maddocks, 2018; McGlynn & Rackley, 2017; Walker & Sleath, 2017). However, this term fails to recognize the different motives that might lead people to disseminate someone else’s sexual pictures without their consent (Maddocks, 2018; Walker & Sleath, 2017). Research has shown that people might disseminate pictures as a joke, to show off, because they find the depicted person attractive, or because they did not consider it to be an issue (Barrense-Dias et al., 2020; Clancy et al., 2020; Eaton et al., 2017). In short, the reasons for the nonconsensual dissemination of sexual pictures are mixed and are not necessarily related to revenge (Clancy et al., 2020; Walker & Sleath, 2017). Importantly, the term “revenge porn” suggests that the targets of the nonconsensual dissemination are to blame, as they were guilty of something that should be punished and that is worthy of revenge (DiTullio & Sullivan, 2019; Maddocks, 2018).

### Image-Based Sexual Abuse

The nonconsensual dissemination of sexual pictures should rather be considered a form of image-based sexual abuse, a category that includes two further forms of aggravated sexting, namely the nonconsensual creation of sexual images and the threat to disseminate sexts (Gassó et al., 2021; Henry et al., 2019; McGlynn & Rackley, 2017; Walker & Sleath, 2017). The nonconsensual dissemination of sexual images can be placed on a continuum of sexual violence, which includes all the sexually coercive and abusive behaviors ranging from catcalling to rape (Kelly, 1988; McGlynn & Rackley, 2017; McGlynn et al., 2021). The nonconsensual dissemination of sexual pictures has indeed been found associated with other forms of sexual abuse, such as dating violence (Frankel et al., 2018; Morelli et al., 2016). Categorizing this phenomenon as a form of sexual abuse reflects the experiences of individuals whose pictures have been distributed, who usually describe the incident as an intrusive violation of their privacy and a breach of trust that had repercussions in terms of their social lives and well-being (McGlynn et al., 2021). As it happens with targets of other forms of sexual abuse, targets of nonconsensual dissemination are also blamed for having been victimized (Eaton & McGlynn, 2020). They are in fact considered guilty of taking the picture in the first place, especially when sharing it for the sake of self-expression or sexual attention from someone who was not their romantic partner (Eaton & McGlynn, 2020; Sciacca et al., 2021; Zvi & Shechory Bitton, 2021). Finally, as it happens with sexual harassment, the seriousness of the nonconsensual dissemination of sexual images is often minimized, as this phenomenon is seen as something funny and harmless by some (Karasavva et al., 2022; Naezer & van Oosterhout, 2021; Powell et al., 2022).

According to a recent systematic review, the nonconsensual dissemination of sexual pictures is quite common among adolescents, with the rates of victimization ranging from 1.5% to 32% depending on different methods of assessment (Walker & Sleath, 2017). However, when disseminating other’s sexual pictures without consent, adolescents might not be aware of engaging in a form of sexual abuse, which is legally prosecuted in some countries (Wolak & Finkelhor, 2011). While navigating a developmental stage characterized by the discovery of nudity and sexuality, teenagers might feel shocked or uncomfortable when confronted with sexual pictures, and therefore might decide to share it with friends to unload the tension or to explore their sexuality (Naezer & van Oostenhout, 2021). In some instances, forwarding someone else’s pictures to a friend might also be a way to share a new experience with someone close and to reinforce the friendship, rather than humiliating the person depicted (Naezer & van Oostenhout, 2021). Furthermore, teenagers might engage in victim-blaming practices because of the inaccurate educational campaigns they witness regarding sexting, that often frame the targets of nonconsensual dissemination as guilty and worthy of blame (Dobson & Ringrose, 2016; Jørgensen et al., 2019).

### The Association Between Image-Based Sexual Abuse and Mental Health

While literature does not focus much on the impact of the nonconsensual dissemination of sexual images on adolescent mental health, media reports show several stories of teenagers who have attempted or committed suicide after their sexual pictures were disseminated without consent (Ankel, 2018; Meacham, 2018; Stevens, 2019). Not surprisingly, the available literature confirms this trend by showing that being a target of aggravated sexting can have detrimental consequences on one’s mental health, that are similar to the ones experienced by targets of face-to-face forms of sexual abuse, such as sexual assault and sexual harassment (Bates, 2017; Mandau, 2021). Targets of various forms of image-based sexual abuse (i.e., the nonconsensual dissemination and creation of sexual images and sextortion) display high levels of stress, depression, anxiety, suicidality, and poor coping strategies, such as substance use (Champion et al., 2022; Patel & Roesch, 2022). Studies on targets of nonconsensual dissemination of sexual images have mostly focused on adult samples, showing that targets report poor mental health, with anxiety, depression, post-traumatic stress disorder, low self-esteem, self-harm, and somatic symptoms being the most common experiences (Bates, 2017; Campbell et al., 2022; Eaton et al., 2017; Eaton & McGlynn, 2020; Gassó et al., 2020; 2021; Huber, 2023).

#### Internalizing problems

From an interpersonal perspective, adult targets (especially women) report experiencing trust issues toward potential partners and fear of starting new relationships (Bates, 2017; Campbell et al., 2022). In some instances, the extreme levels of social anxiety inhibit them from leaving the house, leading to the rupture of social boundaries and to social isolation (Campbell et al., 2022; Eaton & McGlynn, 2020). The social rupture is further enhanced by the deletion of their social media accounts, in which some targets engage in the attempt not to be reminded of the nonconsensual dissemination of their own sexual images (Bates, 2017). Being worried about the distribution of sexual images is indeed common among the targets of this type of victimization, who live in constant anxiety as they are not sure about whether their pictures are still circulating on the Internet (Huber, 2023). Studies sampling adolescents found results comparable to the research studies conducted with adult samples. In fact, the limited evidence on adolescent targets of nonconsensual dissemination and/or other forms of image-based sexual abuse shows that these adolescents display anxiety, depression, suicidality, and self-harm (Frankel et al., 2018; Gámez-Guadix et al., 2022; Mandau, 2021; Pampati et al., 2020).

#### Self-esteem

Research exploring the association between self-esteem and the nonconsensual sharing of intimate images in adolescent samples is lacking. However, research studies investigating similar phenomena (e.g., revenge porn) in samples of adult women showed that having one’s own sexual images shared online without consent has a negative impact on the target’s self-esteem and self-confidence (Aborisade, 2022; Bates, 2017). Technology-facilitated sexual violence, which encompasses the sharing of sexual photos without permission, has also been found to be associated with low self-esteem among young adults (Echevarria et al., 2022). Despite these findings pertaining to samples of young adults, more research exploring the association between self-esteem and the nonconsensual dissemination of sexual images among adolescents is needed. Investigating this association among adolescents is paramount for at least two reasons. First, this phenomenon may be particularly detrimental during adolescence, when young people’s self-esteem is dependent on being popular and liked among their peers (de Bruin & van den Boom, 2005). Moreover, adolescents may feel ashamed and helpless, while not having the coping resources needed to deal with having their nude pictures disseminated against their will (Wachs et al., 2021). This, in turn, may be associated with depression and low self-esteem, which as shown above, have not yet been examined among adolescents who are targeted by the nonconsensual image sharing.

Another important reason for investigating the above pertains to existing research failing to distinguish between distinct forms of online sexual abuse (Gámez-Guadix & Incera, 2021). For example, previous research collapsed the nonconsensual dissemination of sexual images together with other online sexual victimization experiences, including sextortion and the nonconsensual creation of sexual images (e.g., Champion et al., 2022; Mandau, 2021; McGlynn et al., 2021; Patel & Roesch, 2022). However, a focus on the nonconsensual dissemination of sexual images as a phenomenon with distinctive features is needed to uncover its specific contribution to self-esteem and depression among adolescents.

### Gender Differences

Previous studies have focused on the gender differences regarding the prevalence of the nonconsensual dissemination of sexual images. Results are mixed, with some studies showing that this type of victimization is more common among women (Eaton et al., 2017; Karasavva & Forth, 2021) and others finding no differences (Henry et al., 2019; Pedersen et al., 2022). Most of the research studies investigating the nonconsensual dissemination of sexual images have been conducted with samples of adults and young adults. However, the available evidence on adolescents shows that boys engage in more nonconsensual sharing of sexual images compared to girls, and that they usually disseminate pictures of girls (Wachs et al., 2021). Qualitative studies showed that teenagers’ norms and taboos regarding sexting are highly gendered, in that adolescents are more likely to evaluate girls’ pictures as “sexual” compared to boys’ pictures (Naezer & van Oosterhout, 2021). These gendered evaluations of sexual images have been suggested to make girls more likely to be targeted by the nonconsensual image sharing, as girls’ pictures could be judged as more “interesting” to share (Naezer & van Oosterhout, 2021). Based on the available evidence, it is not clear whether women and girls are more frequently targeted by the nonconsensual dissemination compared to boys and men. Nevertheless, it seems to be well established that women undergo blame more often than men for being victimized (Dobson & Ringrose, 2016; Mandau, 2021; Walker & Sleath, 2017; Zvi, 2021).

#### Sexual scripting theory

The tendency to blame women for being victimized more often than men might also be explained by gendered sexting scripts (Symons et al., 2018). In particular, such scripts envisage that in heterosexual relationships, boys are the ones who ask girls for sexual pictures, while girls are pressured to produce pictures. Simultaneously, girls are also expected to be the gatekeepers and resist boys’ requests (Meehan, 2022; Ricciardelli & Adorjan, 2019; Symons et al., 2018). Thus, girls undergo criticism either for sending their self-made sexual pictures (e.g., “slut”) or for not sending them (e.g., “prude”; Lippman & Campbell, 2014). Even when pictures are distributed around without girls’ consent, they will be blamed for not having resisted the request in the first place (Meehan, 2021; Ricciardelli & Adorjan, 2019). Instead, boys who share intimate images without consent will not be accused of violating girls’ privacy, as such behavior is in line with gender norms (Meehan, 2021; Walker & Sleath, 2017). While some studies found that men in particular are blaming women for their sexual conduct (Zvi, 2021; Zvi & Shechory Bitton, 2021), others found that women tend to blame other women who are targeted by the nonconsensual dissemination, possibly to distance themselves from the targets and to hold the belief that they will not be targeted as long as they avoid doing anything that could “provoke” the victimization (Meehan 2021; 2022; Zvi & Shechory Bitton, 2021). This tendency has been documented in adolescent samples, whereby girls tended to blame other girls who were targeted by the nonconsensual dissemination and even participated in the perpetration by forwarding the sexual images, insulting the target, and spreading rumors (Meehan 2021, 2022). Existing research has shown that the consequences of nonconsensual image sharing are not as serious when the images disseminated without consent depict boys. In these instances, the shared pictures might not be a source of stigma and criticism toward the targeted boy; instead, they may produce a humorous effect and could even be laughed off (Mandau, 2021; Ringrose et al., 2022). While saving boys from the stigma, this reaction implies that the consequences of nonconsensual dissemination of sexual pictures are not so serious for boys (Zvi, 2021). However, it must be noted that traditional gender roles, and societal perception of male sexuality, may prevent men from self-identifying as victims of sexual victimization (Depraetere et al., 2020). In this respect, existing research studies have shown that similar to girls, boys might undergo serious psychological damage when their nude pictures are shared without consent (Zvi, 2021). Therefore, it is paramount to look at the experiences of both boys and girls when investigating the mental health problems associated with the nonconsensual dissemination of sexual pictures.

### Sexual Orientation Differences

Sexting seems to be a more common and acceptable practice among lesbian, gay, bisexual, transgender, and queer (LGBTQ+) adolescents. Indeed, existing research showed that LGBTQ+ young people engage in different sexting behaviors more frequently compared to their heterosexual peers (Foody et al., 2021; Hertlein et al., 2015; Morelli et al., 2016; Needham, 2021; Van Ouytsel et al., 2019; Wachs et al., 2021). Besides the motives that lead adolescents to engage in sexting, LGBTQ+ adolescents might be also pushed by the need to build sexual and romantic relationships in a safe space such as the Internet, where they are less exposed to the risk of stigma and discrimination (Ybarra & Mitchell, 2014; Wachs et al., 2021). Furthermore, LGBTQ+ people feel safer in terms of opening up about their sexual orientation online, and therefore are more visible to potential same-sex partners (Needham, 2021). Moreover, LGBTQ+ adolescents might find it easier to receive feedback about their body image from people they are flirting with online (Needham, 2021). Besides engaging more in consensual sexting, LGBTQ+ adolescents are also more often the targets of aggravated sexting when compared to their heterosexual peers, as they are more frequently pressured to sext (Van Ouytsel et al., 2019). With regards to the nonconsensual dissemination of sexual images, results are mixed: While LGBTQ+ adults are more at risk of being a target compared to heterosexuals (Henry et al., 2019; Karasavva & Forth, 2021; Lenhart et al., 2016), research on adolescents found mixed results, with some studies indicating that LGBTQ+ adolescents are more victimized (Priebe & Svedin, 2012) and other studies not showing such difference (Pedersen et al., 2022).

### The Present Study

This study is aimed to examine the nonconsensual dissemination of sexual images in a sample of adolescents. Given the mixed findings in the existing literature, it is fundamental to keep in mind any gender and sexual orientation differences when investigating this phenomenon. Therefore, this study explored whether the prevalence of nonconsensual dissemination of sexual images varies based on gender and sexual orientation. Moreover, this study aimed to explore the associations between enduring the nonconsensual sharing of sexual images and adolescent mental health, with a focus on self-esteem and depression. The following hypotheses were formulated:

H1: Girls and LGB+ adolescents were more often expected to be the targets of nonconsensual dissemination of sexual images compared, respectively, to boys and heterosexual adolescents.
H2: The victimization from the nonconsensual dissemination of sexual images was expected to be positively associated with depression and negatively associated with self-esteem.

## Method

### Participants

The survey was completed by 728 high school students in Sweden. The only inclusion criterion for this study was being a high school student aged between 12 and 19 years. No exclusion criteria were applied in this study. The sample comprised of 50.4% girls, 46.4% boys, and 1% nonbinary people, while 2.2% of participants did not report their gender. Age ranged from 12 to 19 years (*M* = 14.35, *SD* = 1.29, median = 14). The majority of participants identified as heterosexual (85.6%), followed by bisexual (8.1%), pansexual (1.9%), lesbian (1.3%), asexual (1%), and gay (0.5%), while 1.7% of participants reported their sexual orientation as “other.”

### Procedure

Data were collected between October 2019 and May 2020 in Sweden. Students were recruited in two ways. First, a campaign advertising the study was launched on the authors’ institution website. Second, letters were sent to the schools of the authors’ network providing information about the study and consent forms. School principals interested in the project contacted the researchers and provided their consent. Subsequently, information and consent forms were sent to parents of students younger than 15 years. In line with the Swedish legislation, parental consent is not needed for adolescents aged 15 years or older. Thus, respondents aged 15 years were only asked to sign an assent form. In total, five schools participated in the study. Data were collected during school hours through esmaker (i.e., an online survey platform through which the online survey was created and distributed). Before completing the survey, students were informed that their answers were anonymous and that they could quit the survey at any time. Respondents could choose to complete the questionnaire in either English or Swedish. The study was approved by the Regional Ethical Review Board (2018/1803-31/5).

### Instruments

#### Demographics

The survey included a section for collecting information on respondents’ age, gender, and sexual orientation.

#### Nonconsensual dissemination of sexual images

One item was administered to assess respondents’ experiences of nonconsensual dissemination of sexual images: “Has anyone ever shared a sexual image(s) of you (with other people) without your consent or permission?” Answers were given on a 4-point Likert scale ranging from 1 = never to 4 = many times.

#### Depressive symptoms

The short version of the Moods and Feelings Questionnaire (Angold & Costello, 1987) includes 13 items assessing respondents’ depression levels. Participants were asked to indicate how they had felt in the previous 2 weeks. Answers were given on a 3-point Likert scale (1 = not true, 2 = sometimes, and 3 = true). Sample items were “I felt miserable or unhappy” and “I didn’t enjoy anything at all.” A total score was computed for each participant by averaging their answers across the items so that higher scores identified higher levels of depressive symptoms (*α* = .93).

#### Self-esteem

Participants’ self-esteem was assessed through the Rosenberg’s Self-Esteem Scale (Rosenberg, 1965). Participants were given a list of 10 statements and for each they were asked to provide their agreement on a 4-point Likert scale (1 = strongly agree; 4 = strongly disagree). Sample items were “I feel that I have a number of good qualities” and “I feel I do not have much to be proud of.” A total score was computed by reverse scoring the items indicating a positive attitude toward the self, and by averaging respondents’ answers across the items so that higher scores indicated higher levels of self-esteem (*α* = .88).

### Data Analysis

Data were analyzed using SPSS 27 version. Respondents who identified themselves as nonbinary or who did not report their gender (N = 22) were coded as missing values, as the sample size for this subgroup was too small to be analyzed separately. Furthermore, respondents who identified as lesbian, gay, bisexual, pansexual, asexual, and “other” (LGB+) were collapsed into one group, as the frequencies of each sexual orientation were too low to be used for statistical analyses. Missing data were equivalent to 5.2% and they were handled using listwise deletion. Descriptive statistics and correlations were performed for all the study variables.

To answer H1, frequencies were calculated for the nonconsensual dissemination item based on respondents’ gender and sexual orientation. Following this, two chi-squared tests were performed to test for any significant differences in terms of enduring nonconsensual dissemination of intimate images among respondents based on their gender and sexual orientation. The response options in the nonconsensual dissemination of intimate images (“once,” “a few times,” and “many times”) were combined into one single value, due to the frequencies of each response option being too few to perform the chi-squared analysis. Therefore, the resulting nominal variable had two categories: respondents who had never been a target of nonconsensual dissemination (*N* = 645) and respondents who had been a target of nonconsensual dissemination at least once (*N* = 45).

To answer H2, two hierarchical multiple regressions were performed to assess the relationship between being a target of nonconsensual dissemination and depression and self-esteem. Being a target of nonconsensual dissemination of intimate images was used as the predictor variable, and depression and self-esteem were treated as dependent variables. Age, gender, and sexual orientation were used as control variables. Our choice to include the background variables (demographics) in the first step is based on existing research showing that demographic factors including age, gender, and sexual orientation play a role in relation to self-esteem and depression (McDonald, 2018; Moksnes & Johansen Reidunsdatter, 2019; Orth et al., 2018). The victimization from nonconsensual dissemination was included in the second step to test for its effect on depression and self-esteem over and above the role played by well-established variables (i.e., our control variables; Field, 2013). Before running the hierarchical multiple regressions, the assumptions of multicollinearity and normality were tested. For the first, the variance inflation factor values of the predictor variables were calculated, resulting in 1.037 for age, 1.025 for gender, 1.052 for sexual orientation, and 1.015 for being a target of nonconsensual dissemination. Such values indicate that the variables were not correlated; thus, multicollinearity was not detected across the independent variables (Alin, 2010). To test for the assumption of normality, a Shapiro–Wilk test was run for the residuals of each regression. The test proved significant for both the residuals of the regression for depression, *W*(600) = .97, *p* < .001, and the residuals of the regression for self-esteem, *W*(600) = .99, *p* < .001, indicating that the assumption of normality was violated. However, multiple regression is still robust when the residuals are not normally distributed, when performed with large sample sizes (Ghasemi & Zahediasl, 2012); hence, we proceeded to perform the regressions.

## Results

### Descriptive Analyses and Bivariate Correlations

Means and standard deviations for age, nonconsensual dissemination of sexual images, depressive symptoms, and self-esteem are provided in Table 1. The average score for each variable was below the midpoint, except for self-esteem, indicating that, on an average, participants did not have frequent experiences of nonconsensual dissemination and had low levels of depressive symptoms while they had a high self-esteem. The correlation matrix shows that being a target of nonconsensual dissemination of sexual images correlated positively with age, indicating that this experience increases as adolescents grow older. Furthermore, experiencing the nonconsensual dissemination of sexual images was associated positively with depressive symptoms and negatively with self-esteem, indicating that being a target of this phenomenon is associated with higher depression levels and lower self-esteem.

**Table 1.** Descriptive Statistics and Bivariate Correlations for All Study Variables.

Variables	*M* (*SD*)	1	2	3	4
Age	14.35 (1.29)	—			
Nonconsensual dissemination	1.09 (0.39)	.14**	—		
Depressive symptoms	1.66 (0.55)	.13**	.16**	—	—
Self-esteem	2.84 (0.67)	−.11**	−.11**	−.66**	—

** *p* < .01.

### H1: Differences Based on Gender and Sexual Orientation

Frequencies of the nonconsensual dissemination item were calculated for the whole sample, as well as by gender and sexual orientation (Table 2). To answer H1, two chi-squared tests were performed to compare, respectively, boys and girls with heterosexual and LGB+ respondents in terms of their experiences with the nonconsensual dissemination of sexual images. The first chi-squared test considered the groups of boys and girls, while the second chi-squared test considered the groups of heterosexuals and LGB+ participants. The chi-squared test for gender did not yield any significant results. The chi-squared test for sexual orientation showed that a significantly higher proportion of LGB+ participants than expected reported being a target of nonconsensual dissemination of sexual images at least once in their life, χ^2^(1) = 8.18, *p* = .008. Cramér’s V statistic was significant (*p* = .008) with a value of .11, indicating a small effect size.

**Table 2.** Frequencies for Being a Target of Nonconsensual Dissemination Divided by Gender and Sexual Orientation.

Groups	Never *N* (%)	At Least Once *N* (%)
Females	315 (92.6%)	25 (7.4%)
Males	298 (95.2%)	15 (4.8%)
Heterosexuals	507 (94.1%)	32 (5.9%)
LGB+	78 (85.7%)	13 (14.3%)
Total	645 (93.5%)	45 (6.5%)

LGB *=* lesbian, gay, bisexual.

### H2: Non-Consensual Dissemination, Depression, and Self-Esteem

To answer H2, two hierarchical multiple regressions were conducted, with depression and self-esteem as the outcome variables. Predictor variables were the same in both regressions. In the first step, age, gender, and sexual orientation were entered as control variables, while the nonconsensual dissemination item was entered in the second step. The first regression model including depression as an outcome variable showed that girls (*B* = −.41, *t* = −10.08, *p* < .001; Table 3), LGB+ adolescents (*B* = .13, *t* = 2.20, *p* = .028), and older adolescents (*B* = .03, *t* = 2.10, *p* = .036) experienced higher levels of depression. After controlling for the role of demographic variables, results showed that being a target of nonconsensual dissemination of sexual images was related to higher levels of depressive symptoms (*B* = .16, *t* = 2.86, *p* = .004). The second regression model, which included self-esteem as an outcome, showed that boys (*B* = .46, *t* = 8.97, *p* < .001), heterosexual adolescents (*B* = −.21, *t* = −2.74, *p* = .006), and younger adolescents (*B* = −.04, *t* = −2.18, *p* = .030) displayed higher levels of self-esteem. However, no significant results were yielded for experiencing the nonconsensual dissemination of sexual images.

**Table 3.** Hierarchical Multiple Regression Analysis Predicting Depressive Symptoms’ Scores.

Predictors	*R*^2^ (Δ*R*^2^)	*B*	SE	*t*_(599)_	95% CI
Step 1	.17 (.17***)	1.35***	0.23	5.81	[0.90, 1.81]
Gender (male = 1)		−0.41***	0.04	−10.08	[−0.49, −0.33]
Sexual orientation (LGB+ = 1)		0.13*	0.06	2.20	[0.01, 0.25]
Age		0.03*	0.02	2.10	[0.00, 0.07]
Step 2	.18 (.01**)	1.23***	0.24	5.23	[0.77, 1.69]
Gender (male = 1)		−0.40***	0.04	−9.96	[−0.48, −0.32]
Sexual orientation (LGB+ = 1)		0.13*	0.06	2.08	[0.01, 0.25]
Age		0.03	0.02	1.88	[−0.00, 0.06]
Nonconsensual dissemination		0.16**	0.06	2.86	[0.05, 0.27]

LGB = lesbian, gay, bisexual.

* *p* < .05. **p < .01. ***p < .001.

## Discussion

The goal of this study was to investigate the prevalence of the nonconsensual dissemination of sexual images among adolescents based on gender and sexual orientation, and its association with depression and self-esteem. Findings point to sexual orientation as an important variable to take into consideration when investigating adolescents’ experiences of nonconsensual dissemination of intimate images. Moreover, the study findings highlight the importance of looking at the mental health correlates of these experiences. In the text that follows, we discuss the specifics of the study findings, by addressing each hypothesis individually.

### Gender and Sexual Orientation Differences

Our findings show that overall, 6.5% of participants had their sexual pictures shared without their consent at least once over the course of their lives. This is in line with previous studies that found a similar prevalence when investigating this form of victimization among adolescents (Gámez-Guadix et al., 2022; Pampati et al., 2020; Pedersen et al., 2022). Specifically, the victimization rates in this were 7.4% for girls and 4.8% for boys. The association between gender and being a target of nonconsensual dissemination was not significant. On one hand, such result is consistent with findings from previous studies on the nonconsensual dissemination among adults (Gámez-Guadix et al., 2015), and from studies on image-based sexual abuse among adolescents (Pedersen et al., 2022). However, on the other hand, our findings are in contrast with previous research that did detect significant gender differences among both adults and adolescents (Karasavva & Forth, 2021; Pampati et al., 2020). Given these mixed results, and based on the findings of this study, we may assume that girls and boys could be equally targeted by the nonconsensual dissemination of sexual images, although the associations between these experiences and mental health outcomes do differ significantly based on gender (Henry et al., 2019; Mandau, 2021; Walker & Sleath, 2017).

Conversely to gender, a significant relationship between being a target of nonconsensual dissemination of sexual images and sexual orientation, with 14.3% of sexual minority adolescents having their nudes shared without consent at least once in their life, as opposed to the 5.9% of heterosexual adolescents. This result is in line with previous studies on adolescents and adults (Foody et al., 2021; Henry et al., 2019; Karasavva & Forth, 2021; Lenhart et al., 2016; Priebe & Svedin, 2012), while being in contrast with other research that did not find any significant differences between heterosexual and LGB+ teenagers (Pedersen et al., 2022). A possible explanation is that LGB+ adolescents sext and use the Internet more frequently than their heterosexual peers; moreover, they might explore their sexuality and find potential partners online (Morelli et al., 2016; Needham, 20201; Van Ouytsel et al., 2019; Wachs et al., 2021), which could increase their risk of being exposed to victimization experiences. Based on previous research, it is likely that sexual minority adolescents who are targets of nonconsensual dissemination of sexual images endure multiple forms of abuse, including other forms of aggravated sexting (Van Ouytsel et al., 2019), and online harassment in general (Backe et al., 2018; Escobar-Viera et al., 2018; Llorent et al., 2016; Sterzing et al., 2019). Being a target of multiple forms of victimization is known as polyvictimization, and it has been shown to have cumulative adverse mental health outcomes (Kassing et al., 2021; Powell et al., 2018). This, in turn, calls for mental health support services and intervention programs specifically targeted for sexual minority adolescents who endure the nonconsensual dissemination of intimate images.

### Association with Depression and Self-Esteem

This study findings show that being a target of nonconsensual dissemination of sexual images is associated with higher depression, while it is not associated with self-esteem levels. Such results are partially in line with previous research conducted with both adolescent (Frankel et al., 2018; Gámez-Guadix et al., 2022) and adult samples, indicating that the nonconsensual dissemination of sexual images was associated with high levels of depression (Bates, 2017; Champion et al., 2022; Gassó et al., 2020; Huber, 2023; Patel & Roesch, 2022). Targets of nonconsensual dissemination (girls in particular) tend to blame themselves for their victimization: They feel responsible for what happened, thinking that they behaved in a naïve manner and that they made a “bad choice” when they produced and sent their self-made sexual picture to the perpetrator (Mandau, 2021). Such feelings of self-blame, together with feelings of shame, might increase their depressive symptoms (Alix et al., 2020), while preventing them from seeking help (Weiss, 2010). As a result, adolescents might not be able to receive the emotional support that they need, which could further exacerbate their symptoms of depression. Besides being victimized through the nonconsensual dissemination of sexual images, targets might be exposed to secondary victimization too (Veronica & Di Giacomo, 2022). More specifically, the target might not be taken seriously as the nonconsensual dissemination is sometimes trivialized and excused by other people. Previous research has shown that some people (both adolescents and adults) might hold false beliefs in relation to the nonconsensual dissemination of sexual images, which are aimed at downplaying the abuse. Examples are believing that the nonconsensual dissemination is inevitable, that it is harmless, and that the person who sends the picture is partially responsible for the dissemination (Clancy et al., 2020; Powell et al., 2019). There are also some scenarios where the nonconsensual dissemination of sexual images is considered more acceptable, for instance when the nonconsensually distributed pictures are shared outside of a romantic relationship (Naezer & van Oosterhout, 2021). In this case, the target is seen as someone who was “seeking attention” and who therefore deserves to be blamed for having shared their pictures in the first place (Naezer & van Oosterhout, 2021; Sciacca et al., 2021). Therefore, targets of nonconsensual dissemination might be diminished, made fun of, insulted, humiliated, physically harmed, and harassed by others because they sent the sexual picture in the first place (Gassó et al., 2021). This experience is particularly common among girls (Dobson & Ringrose, 2016; Goblet & Glowacz, 2021). Experiencing secondary victimization might exacerbate targets’ depressive symptoms and global mental health (Gassó et al., 2021; Goblet & Glowacz, 2021). It should be noted that experiencing mental health issues and enduring secondary victimization is common among both targets of digital sexual abuse and among those who are targeted by offline forms of sexual violence. This, in turn, makes the outcomes of these experiences comparable despite the means (online vs. offline) through which these forms of abuse are perpetrated (Bates, 2017; Eaton & McGlynn, 2020; Mandau, 2021; McGlynn et al., 2021; Zvi & Shechory Bitton, 2021).

In contrast with findings from previous literature, this study did not find any association between the nonconsensual dissemination of sexual images and self-esteem. Targets of nonconsensual dissemination usually experience a decrease in their self-esteem levels, as the loss of control they feel over those who see their private pictures has a negative impact on their self-confidence and self-esteem (Bates, 2017; Huber, 2023). It could be that adolescents in this study managed to cope with the nonconsensual dissemination of sexual images, enough for it not to interfere with their self-esteem levels. Alternatively, certain specific aspects of self-esteem (as opposed to self-esteem as a global construct), including body self-esteem could play a more prominent role in relation to the nonconsensual dissemination of sexual images. In this respect, research studies looking at specific components of body self-esteem found a negative association between online sexual victimization (sextortion) and body satisfaction (Tamarit et al., 2021).

### Strengths, Limitations, and Future Directions

This study represents a contribution to the existing literature on aggravated sexting. We focused specifically on the nonconsensual dissemination of sexual images separating it from other forms of image-based sexual abuse, such as the nonconsensual creation of sexual images and sextortion. This distinction is not always present in the literature, which often collapses different forms of victimization (Gámez-Guadix & Incera, 2021). Second, we sampled adolescent participants, as the literature on the mental health of adolescent targets of nonconsensual dissemination is not very widespread, despite this age group being at a higher risk for this type of victimization (Walker & Sleath, 2017). In particular, our sample included younger adolescents (i.e., 12–15 years), which is important considering that sexting literature often lacks a focus on this age group (Van Ouytsel et al., 2018). Finally, we examined gender and sexual orientation differences in the prevalence of the nonconsensual dissemination of sexual images, as previous research has led to mixed results.

Despite such contributions, this study displays some limitations as well. First, the cross-sectional study design prevents us from drawing any conclusions regarding the causality of the observed associations. It is suggested for future research to uncover the longitudinal association between the nonconsensual dissemination of sexual images and mental health variables. The lack of a significant association between the nonconsensual dissemination of intimate images and self-esteem could be related to the cross-sectional design of this study. More specifically, the negative impact of nonconsensual dissemination on self-esteem might unfold over time, though this remains to be investigated in future research. Second, only a small number of respondents was targeted by the nonconsensual sharing of intimate images, which constitutes a further limitation of this study. This small subsample might have affected the statistical power of our analysis, preventing us from finding both a significant association between being a target of nonconsensual dissemination and self-esteem, and a significant difference between boys and girls in terms of being targets of nonconsensual dissemination. Such risk of incurring a type II error might be overcome by future research by expanding the sample though diversifying the sources and strategies of recruitment (Gerassi et al., 2017). Adopting sampling strategies such as targeted sampling (i.e., recruiting respondents based on specific attributes) may help to reach “hidden populations,” such as young people who are targeted by the nonconsensual sharing of intimate images (Dusek et al., 2015). For example, it may be helpful to collect data through organizations (e.g., counseling and psychological support services) that could come into contact with young people who are targeted by this phenomenon. Third, participants’ victimization was assessed only through one ad hoc item, which, unfortunately, cannot be tested for validity and reliability. Finally, this study adopted a convenience sample including a minority of LGB+ participants. Future research should aim at increasing the LGB+ subsample by specifically recruiting sexual minority adolescents.

### Practical Implications

The nonconsensual dissemination of sexual images is a phenomenon that needs to be tackled as it is related to poor mental health among adolescents. Prevention programs exist in schools, but their approach might risk being counterproductive. Existing programs usually try to persuade young people not to engage in sexting at all while focusing on the responsibilities of the target of the nonconsensual dissemination, claiming that they should have prevented the incident by not sending the sexual pictures in the first place (Dobson & Ringrose, 2016; York, 2021). Such victim-blaming approach could lead adolescents to blame the target while further exacerbating the target’s depressive symptoms (Dobson & Ringrose, 2016; Gassó et al., 2021; Goblet & Glowacz, 2021). Furthermore, teaching adolescents to abstain from sexting prevents them from exploring their sexuality and deprives them of their sexual agency (Döring, 2014; Patchin & Hinduja, 2019). Sex education programs should rather teach adolescents to make ethical decisions when it comes to intimacy and relationships, including sexting. Adolescents should be made aware that the nonconsensual dissemination of sexual images is a form of abuse that can seriously harm the target, and sanctions should be secured for those who distribute someone’s sexual picture without consent, following the restorative justice approach (Ojeda & Del Rey, 2022; Rackley et al., 2021). With regards to sexual orientation, sex education is often heteronormative and not inclusive of the experiences of LGB+ adolescents: Sexual and gender minority issues are rarely mentioned, and even less so in combination with sexual abuse (MacAulay et al., 2021). Such topics should be thoroughly covered in sex education modules, and specific educational activities should be carried out concerning the risk of sexual abuse among sexual minority adolescents, as they are at higher risk for being a target of nonconsensual dissemination (Ojeda & Del Rey, 2022). Finally, a dedicated support system should be provided to targets of nonconsensual dissemination. School counselors and specifically appointed school staff members should be available for adolescents to report the nonconsensual dissemination incidents (Barrense-Dias et al., 2020; Ojeda & Del Rey, 2022). Digital tools could also be employed in support for the victimized adolescents, as they are also available outside school hours; for instance, chatbots have proved to be useful in giving information on how to deal with the nonconsensual dissemination as well as in providing emotional support to the targets without blaming them (Maeng & Lee, 2022).

## Conclusions

The nonconsensual dissemination of sexual images has been poorly investigated in adolescent samples. This study shows the importance of taking into consideration adolescent background including sexual orientation, when investigating the nonconsensual sharing of intimate images. Moreover, this study adds new knowledge to the existing literature by showing that disseminating sexual images without consent is associated with depressive symptoms among young people. The lack of a significant association between nonconsensual sharing of intimate images and self-esteem warrants further investigation. A more fine-grained analysis of the distinct self-esteem components may elucidate their associations with the nonconsensual sharing of intimate images in future research. Although the cross-sectional design prevents us from drawing any inferences, the findings of this study may speak of the pervasiveness of the nonconsensual sharing of intimate images in terms of adolescent mental health. Simultaneously, findings of this study call for targeted evidence-based programs aimed at preventing mental health problems associated with the nonconsensual dissemination of intimate images. Importantly, findings point to the need for prevention and intervention programs to overcome heteronormative discourses while addressing the needs of adolescents with different sexual orientations.
